# Low temperature CO oxidation catalysed by flower-like Ni–Co–O: how physicochemical properties influence catalytic performance†

**DOI:** 10.1039/c7ra12635b

**Published:** 2018-02-12

**Authors:** Yunan Yi, Pan Zhang, Zuzeng Qin, Chuxuan Yu, Wei Li, Qiuju Qin, Bin Li, Minguang Fan, Xin Liang, Lihui Dong

**Affiliations:** Guangxi Key Laboratory of Petrochemical Resource Processing and Process Intensification Technology, School of Chemistry and Chemical Engineering, Guangxi University Nanning 530004 P. R. China binli@gxu.edu.cn donglihui2005@126.com; Jiangsu Key Laboratory of Vehicle Emissions Control, Centre of Modern Analysis, Nanjing University Nanjing 210093 P. R. China; Guangxi Colleges and Universities Key Laboratory of Applied Chemistry Technology and Resource Development, Guangxi University Nanning 530004 P. R. China

## Abstract

In this work, mesoporous Ni–Co composite oxides were synthesized by a facile liquid-precipitation method without the addition of surfactant, and their ability to catalyse a low temperature CO oxidation reaction was investigated. To explore the effect of the synergetic interaction between Ni and Co on the physicochemical properties and catalytic performance of these catalysts, the as-prepared samples were characterized using XRF, XRD, LRS, N_2_-physisorption (BET), SEM, TEM, XPS, H_2_-TPR, O_2_-TPD and *in situ* DRIFTS characterization techniques. The results are as follows: (1) the doping of cobalt can reduces the size of NiO, thus massive amorphous NiO have formed and highly dispersed on the catalyst surface, resulting in the formation of abundant surface Ni^2+^ ions; (2) Ni^2+^ ions partially substitute Co^3+^ ions to form a Ni–Co spinel solid solution, generating an abundance of surface oxygen vacancies, which are vital for CO oxidation; (3) the Ni_0.8_Co_0.2_ catalyst exhibits the highest catalytic activity and a satisfactory stability for CO oxidation, whereas a larger cobalt content results in a decrease in activity, suggesting that the amorphous NiO phase is the dominant active phase instead of Co_3_O_4_ for CO oxidation; (4) the introduction of Co can alter the morphology of catalyst from plate-like to flower-like and then to dense granules. This morphological variation is related to the textural properties and catalytic performance of the catalysts. Lastly, a possible mechanism for CO oxidation reaction is tentatively proposed.

## Abstract 2

The flower-like catalyst possesses highly dispersed amorphous NiO and a high concentration of surface oxygen vacancies which are the central points for CO oxidation.

## Graphical abstract

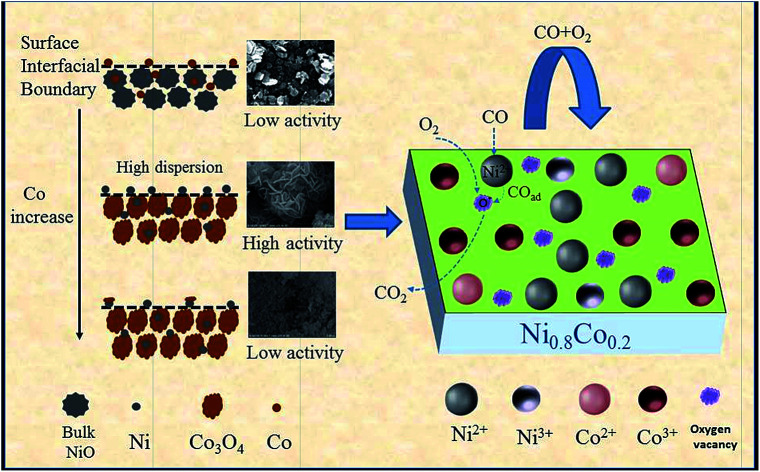

## 1. Introduction

Carbon monoxide is a major atmospheric pollutant. Excessive use of fossil fuel and motor vehicles produces large quantities of exhaust emission, which has resulted in an increase in the level of carbon monoxide (CO) in the atmosphere. CO poses serious threats to humanity, in the form of air pollution and global warming. Catalytic systems play an important role in controlling the elimination of carbon monoxide.^1^ Catalysts containing precious metals (such as Pt, Rh, Pd and Au) are useful in CO oxidation.^2,3^ However, the high cost, low stability, high pollution and scarcity of precious metals limit their use in applications. It is thus imperative to develop a low cost, higher stability and environmentally friendly alternative.^4,5^ Transition metal oxides (such as CuO, CeO_2_, MnO_*x*_ and CoO_*x*_) have received considerable attention as heterogeneous catalysts due in large part to their ability to support effective surface redox reactivity as well as their relative affordability.^6^

Nickel oxide is an earth-abundant transition metal oxide with superior redox property, electrochemical performance and gas sensing property. It is used in many applications such as metallurgy and catalysis and has been used to construct electrodes and gas sensors. Ni-based catalysts are commonly studied for their potential ability to catalyse the dry-reforming reaction on an industrial scale.^7^ In addition, researchers have studied NiO catalysts with various morphologies for CO oxidation, and found that ring-like^8^ and flower-like^9^ NiO demonstrated high activity. NiO–CeO_2_ has recently demonstrated catalytic activity in the CO oxidation and the CO + NO model reactions, due to its high activity and durability. Tang *et al.*^10^ synthesized mesoporous NiO–CeO_2_ catalysts by a KIT-6-templating method, and demonstrated that interfacial NiO is the primary active species for CO oxidation. Cheng *et al.*^11^ combined *in situ* DRIFTS and MS techniques to explore the reaction mechanism of NO removal by CO over a NiO–CeO_2_ catalyst.

As a promising alternative to precious metals, cobalt oxide – particularly Co_3_O_4_, a representative spinel structure transition metal oxide, has been extensively studied and shows very high activity for CO oxidation at low temperatures. Researchers have assigned the effectiveness of Co_3_O_4_ in low-temperature CO catalytic oxidation to the fact that Co_3_O_4_ is the most active transition metal oxide for CO oxidation, as well as its possession of a unique Co^3+^/Co^2+^ redox couple.^2^ For example, Wang *et al.*^12^ obtained Co_3_O_4_*via* a controlled liquid precipitation process without the use of any surfactant or oxidant and found that it exhibited very high activity for CO oxidation at room temperature or even at −78 °C. Zhang *et al.*^13^ used a dispersion–precipitation method to synthesize nanosized Co_3_O_4_ particles with a high activity (and stability) for the catalytic oxidation of carbon monoxide and propane. Moreover, it was deduced that the oxygen species contributes significantly to the enhanced catalytic activity. However, the low temperature CO oxidation activity of Co_3_O_4_ is inhibited by the presence of water, hydrocarbons and NO. Furthermore, their activity was found to decrease during steady-state CO oxidation although in the absence of inhibitors.^14^ It is one of the main problems that limits its practical application. Thus, the need to develop a stable and efficient catalyst is urgent. One way to overcome these deficiencies may be the formation of binary metal oxides. Benjamin Faure *et al.*^15^ report that Co_*x*_Mn_3−*x*_O_4_ spinel oxide catalysts exhibited an outstanding activity for CO and propane oxidation at mild temperatures, which correlates with the high surface area and cobalt concentration of the catalyst. The Co/CeO_2_ (ref. 16 and 17) and Co_3_O_4_–CeO_2_ (ref. 18) catalysts also demonstrate high CO conversion and reasonable stability for the catalytic reaction of CO preferential oxidation and CO oxidation, respectively.

Furthermore, Ni and Co have similar electronic configurations, which likely results in a Ni–Co composite oxides able to demonstrate a synergistic catalytic effect. Ni–Co materials obtained by different synthetic methods have demonstrated modified catalytic performance and stability in various fields including CO and CO_2_ methanation,^19,20^ propane oxidation,^21^ reforming reactions^22,23^ and as an electrode material.^24^ Yu *et al.*^19^ have revealed that the synergetic effect between Ni and Co over bimetallic catalysts can reduce nickel size to enhance the metal particle dispersion and accelerate the activation of adsorbed CO, thereby improving the catalytic activity and coke resistance. Zhang *et al.*^25^ synthesized a Ni–Co bimetallic catalyst *via* a co-precipitation method and found that the Ni–Co bimetallic catalyst demonstrated superior performance in terms of activity and stability compared to other Ni–Me (Me = Fe, Cu and Mn) bimetallic oxides for the carbon dioxide reforming of methane. The superior catalytic performance was attributed to the synergetic effect, good metal dispersion, high metallic surface area, formation of different types of solid solutions, and a strong-metal-support-interaction. In addition, numerous researchers have recognized that the Ni–Co binary oxide shows a strong adsorption capacity for CO. It was inferred that Ni–Co composite oxides could be potential catalysts for CO oxidation at low temperatures. The use of Ni–Co composite oxides for low temperature CO catalytic oxidation has only been reported as follows. Liang *et al.*^26^ prepared a series of Ni–Co bimetal hydroxides nanosheets for CO oxidation and proposed a reaction analysis to explain the synergetic effect in the Ni–Co bimetal oxides system. The synergetic interaction between Ni and Co affecting the catalytic physicochemical properties and activity taking into account the diverse morphologies of the bimetallic oxide catalysts and the catalytic mechanism is worth further elucidation. In addition, the Ni–Co materials reported previous have ordinary morphologies and low specific surface areas, and their methods of preparation are complex.

In the present work, a series of Ni–Co composite oxides with diverse morphologies were prepared *via* a facile liquid-precipitation method, which is cost-effective and low polluting. The flower-like catalyst exhibits high CO conversion at low temperature, and excellent stability, and therefore has much potential to be used practically. The prepared powder catalysts were characterized with XRF, XRD, LRS, N_2_-physisorption, SEM, TEM, XPS, H_2_-TPR, O_2_-TPD, *in situ* DRIFTS and CO oxidation. This study focuses on: (1) investigating the effects of Co doping on textural properties, morphology, chemical composition, redox properties and catalytic performance of NiO; (2) studying the surface structure and structure–activity correlation of the Ni–Co catalysts for low temperature CO oxidation; (3) and analysing the interaction of CO or/and O_2_ over typical samples by *in situ* DRIFTS, to reveal a possible reaction mechanism for CO oxidation.

## 2. Experimental

### 2.1 Catalyst preparation

The Ni–Co composite oxides and the NiO and Co_3_O_4_ were prepared by the liquid-precipitation method without any surfactant. Briefly, an appropriate amount of Ni(NO_3_)_2_·6H_2_O and CO(NO_3_)_2_·6H_2_O were dissolved in deionized water to obtain 2 mol L^−1^ Ni(NO_3_)_2_ and CO(NO_3_)_2_ aqueous solutions. The two aqueous solutions were mixed with constant stirring at an ambient temperature to obtain mixed aqueous solutions of different Ni/Co molar ratios (theoretical ratios Ni/Co = 99 : 1, 95 : 1, 9 : 1, 8 : 2, 7 : 3). Subsequently, excess diluted ammonia was added to these aqueous solutions dropwise (also with vigorous stirring) until the pH reached ∼10, and suspensions were obtained. After further stirring for 4 h and the samples were left to mature for 18 h at room temperature, before the products were collected by centrifugation. The products were washed consecutively with deionized water and absolute alcohol three times, in sequence, before being dried at 80 °C for 12 h. The obtained presoma were grinded fully and calcined in a muffle furnace at 400 °C for 4 h. For simplification, the samples are denoted as Ni_1−*x*_Co_*x*_, for instance, the sample with a theoretical ratio Ni/Co = 99 : 1 is denoted as Ni_0.99_Co_0.1_. For comparison, pure NiO, Co_3_O_4_ and other Ni_0.8_M_0.2_ (where M = Mn, Fe, Zn, Cr) oxides were prepared using the same procedure.

### 2.2 Characterization of catalysts

The bulk chemical compositions of samples were analysed by X-ray fluorescence (XRF), which was performed on an ARL ADVAT'X IntellipowerTM3600 X-ray fluorescence spectrometer.

Power X-ray diffraction (XRD) patterns were obtained with a X'Pert PRO diffractometer (PANalytical, Netherlands) using Cu/Kα radiation (*λ* = 1.54060 Å). The scanning voltage and current were set to 40 kV and 40 mA. The scanning range of 2*θ* is 10° to 80°, with a scan rate of 8° min^−1^.

Laser Raman spectrometer (LRS) was carried out with a Renishaw InVia Reflex Raman spectrometer using an Ar^+^ laser beam. Raman spectra were obtained under an excitation wavelength of 532 nm and a laser power of 5 mW.

N_2_ adsorption–desorption isotherms at 77 K were obtained with a Micrometrics TriStar II 3020 analyser, and the specific surface area and pore distribution were expressed by Brunauer–Emmett–Teller (BET) and Barrett–Joyner–Halenda (BJH) methods from the nitrogen sorption isotherm, respectively.

Scanning electron microscopy (SEM) measurements were performed with a HITACHI S-3400N electron microscope (Hitachi Company, Japan) at 20 kV. Samples for FESEM were suspended in ethanol and dispersed by ultrasonic, and then dropped onto an aluminium sheet.

Transmission electron microscopy (TEM) images were taken on a Tecnai G2 F20 S-TWIN instrument (FEI Company, America) at an acceleration voltage of 200 kV.

X-ray photoelectron spectroscopy (XPS) was performed on an ESCALAB 250Xi multifunctional imaging electron spectrometer (Thermo Fisher Company, America) using monochromatic Al Kα radiation (*hν* = 1486.6 eV) and operating at a power level of 150 W. The electron binding energy was calibrated based on C 1s (284.8 eV). The sample irradiation area and detecting depth were 2 mm × 1 mm and 2–5 nm, respectively. In addition, the peaks have been fitted by the CasaXPS.

H_2_-TPR was performed by a FINESORB-3010 automated chemisorption apparatus (Finetec Corporation). The sample (15 mg) was heated from room temperature to 110 °C under a N_2_ flow of 50 mL min^−1^ (it was kept under these conditions for 1 h prior to the analysis), before being cooled to room temperature in a N_2_ atmosphere and switched to a stream of mixture of H_2_–Ar (7% H_2_ by volume) at 10 mL min^−1^ for 30 min. Later, the temperature was increased from room temperature to 600 °C (10 °C min^−1^). The H_2_ consumption was continuously analyzed using a thermal conductivity detector (TCD).

O_2_-TPD was performed by a FINESORB-3010 automated chemisorption apparatus from Finetec Corporation. Firstly, the sample (100 mg) was heated from room temperature to 200 °C in a He flow of 30 mL min^−1^, which was maintained for 100 min, before the sample was cooled to room temperature (still in an He atmosphere), and then the sample was exposed to a stream of pure O_2_ (10 mL min^−1^) for 30 min. The sample was then exposed to a He flow for 30 min to clear residual oxygen. After that, it was heated from room temperature to 700 °C in He atmosphere at 10 °C min^−1^. The O_2_ consumption was continuously analysed using TCD.

*In situ* Diffusion Reflectance Infrared Fourier Transform (*In situ* DRIFTS) spectra were collected using a Nicolet iS50 FT-IR spectrometer equipped with a MCT detector set at a resolution of 4 cm^−1^ with 32 scans. The catalyst powders were placed in the sample pool and pre-treated with purified N_2_ at 300 °C for 1 h to eliminate any impurities, before being cooled to room temperature while the background spectra of catalysts at diverse target temperatures were collected. Subsequently, the catalyst was exposed to a stream of CO–N_2_ (2% CO by volume) or/and dry air (21% volume O_2_ and 79% volume N_2_) at a rate of 10.4 and 8.2 mL min^−1^, respectively, for 40 min (until saturation has been reached). The DRIFTS spectra of CO and CO + O_2_ were collected at the target temperature from 50 to 150 °C at a heating rate of 5 °C min^−1^. Finally, the results were obtained by removing the corresponding background reference.

### 2.3 Catalytic activity measurements

The activities of the catalysts for CO oxidation were measured under stationary conditions with a feed stream of 1.6% CO, 20.8% O_2_ and 77.6% N_2_. The 50 mg sample (40–60 mesh) was loaded into a quartz tube and pre-treated at 100 °C under a high purity N_2_ flow for 1 h to eliminate impurities. The sample was then cooled to room temperature before turning on the mixture gas. The reaction was carried out under different temperatures (ranging from room temperature to 160 °C) with a space velocity of 30 000 mL h^−1^ g_cat_^−1^. A gas chromatographer (GC7890II, Shanghai Techcomp) equipped with a TCD was used to analyse the outlet gases. The following formula was used to calculate the CO conversion:CO conversion (%) = ([CO]_in_ − [CO]_out_) × 100%

## 3. Results and discussion

### 3.1 Catalytic performance of the as prepared catalysts

**Fig. 1:**
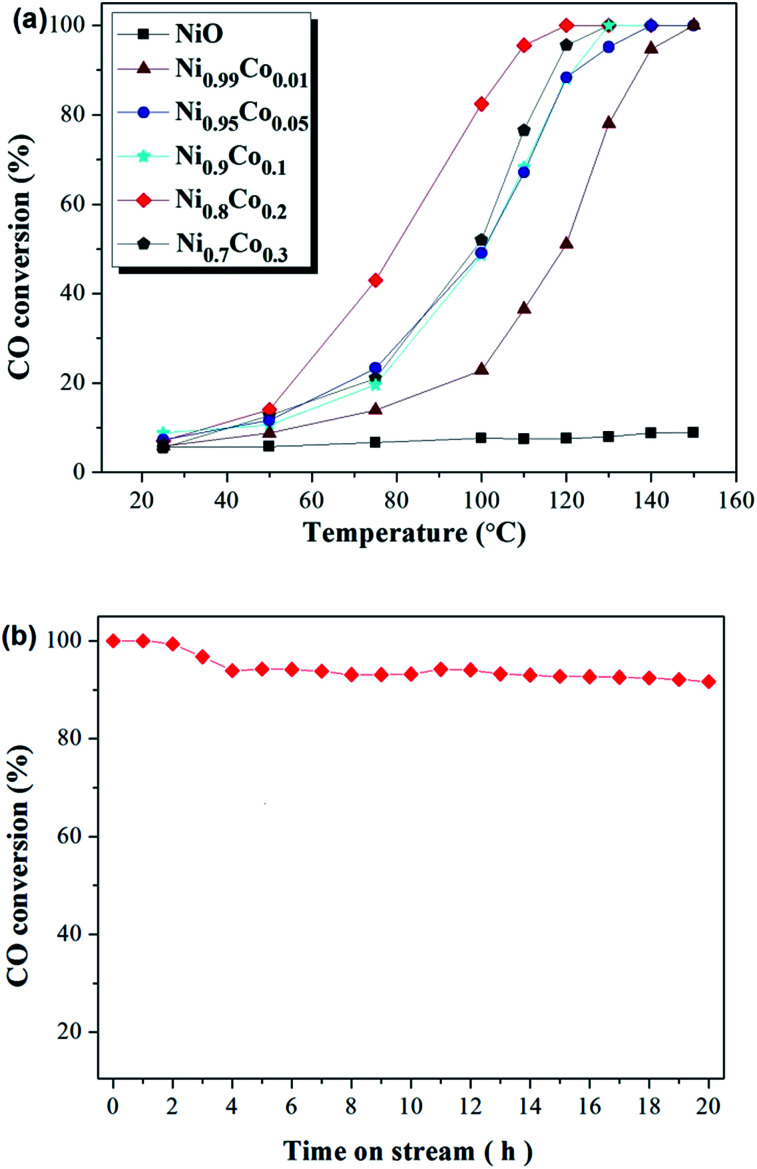
CO conversion (%) over (a) as prepared samples and (b) Ni_0.8_Co_0.2_ sample at 120 °C for 20 h. Feed stream composition: 1.6% CO, 20.8% O_2_ and 77.6% N_2_ by volume.

Catalytic oxidation of CO was conducted to estimate the catalytic performance of the as-prepared catalysts. Fig. 1(a) depicts the activities of Ni–Co, pure NiO and Co_3_O_4_ samples towards CO oxidation. The pure NiO sample displayed very poor CO conversion (less than 10%), and this hardly improved despite an increase in the temperature. The pure Co_3_O_4_ sample also displayed poor CO conversion at low temperatures, but this was observed to increase sharply when the temperature rose above 120 °C. For the Ni–Co composite oxides, all samples show a remarkably increased CO conversion compared with the NiO and Co_3_O_4_ samples, with the complete conversion occurring at temperatures ranging from 120 °C to 150 °C. Moreover, CO conversion increases sharply with temperatures above 50 °C. Interestingly, in comparing Ni_0.99_Co_0.01_ with NiO, despite the minor difference in the content of the two samples, the CO conversion improved greatly. The Ni_0.8_Co_0.2_ sample demonstrated the highest activity, with *T*_50_ at ∼80 °C and *T*_100_ at ∼120 °C. Further increasing the Co content causes the activity to decrease. The activity of the samples follows this order: Ni_0.8_Co_0.2_ > Ni_0.7_Co_0.3_ > Ni_0.9_Co_0.1_ > Ni_0.95_Co_0.05_ > Ni_0.99_Co_0.01_ > Co_3_O_4_ > NiO. The above results demonstrate that doping cobalt into nickel oxide can greatly improve the catalytic activity whilst the Ni/Co ratio influences the catalytic activity. In general, the CO conversions were observed to be different between the first and second runs. For example, the CO conversions results for the two runs of the Ni_0.8_Co_0.2_ sample are shown in the ESI.† As shown in Fig. S1,† the CO conversion of the second run is typically higher than that of the first run for temperatures less than 100 °C.

In addition, catalytic stability is a crucial factor for the heterogeneous catalysis. In order to explore the long-term catalytic stability of the catalysts, the activity of the catalysts was examined at 120 °C over 20 h. The stability of the Ni_0.8_Co_0.2_ catalyst and the corresponding results are shown in Fig. 1(b). It is clear that the powder catalysts and feed gas were able to reach reaction equilibrium after a primary period of 4 h. Furthermore, the Ni_0.8_Co_0.2_ catalyst was found to maintain a high CO conversion, exceeding 90% after 20 h under the reaction conditions. The result demonstrates that the Ni_0.8_Co_0.2_ catalyst has excellent long-term catalytic stability for CO oxidation. In addition, the activity of Ni_0.8_Co_0.2_ is higher than that of the other Ni_0.8_M_0.2_ (M = Mn, Fe, Zn, Cr, Co) composite oxides, with the results for this experiment shown in the ESI (Fig. S2†).

### 3.2 XRF results and textural properties analysis (XRD, LRS, N_2_-physisorption, SEM and TEM)

In order to determine the bulk chemical composition of the Ni–Co samples, XRF was conducted, and the results are shown in Table 1. It can be seen that the actual proportion of Co species is larger than the theoretical ratio for all samples, which may be caused by the loss of nickel during preparation.

**Table 1:** XRF results and textural properties of the as prepared samples

Sample	Atomic ratio Co/(Ni + Co)^a^ (%)	BET surface area (m^2^ g^−1^)	Total pore volume (cm^3^ g^−1^)	Average pore diameter/(nm)	Crystallite size^b^ (nm)
NiO	—	112	0.443	15.891	11.4
Ni_0.99_Co_0.01_	3.41	117	0.369	12.620	10.9
Ni_0.95_Co_0.05_	16.29	135	0.552	16.582	8.2
Ni_0.9_Co_0.1_	29.56	119	0.562	18.840	3.6
Ni_0.8_Co_0.2_	43.76	111	0.551	19.796	2.9
Ni_0.7_Co_0.3_	55.06	83	0.403	19.448	6.0
Co_3_O_4_	—	61	0.180	11.782	18.6

a Calculated by XRF results.

b Determined by the Scherrer equation with the (111) diffraction peak of face-centred cubic phase for NiO, Ni_0.99_Co_0.01_, Ni_0.95_Co_0.05_ and Ni_0.9_Co_0.1_ samples, and with the (311) diffraction peak of spinel phase for Co_3_O_4,_ Ni_0.8_Co_0.2_ and Ni_0.7_ Co_0.3_ samples.

**Fig. 2:**
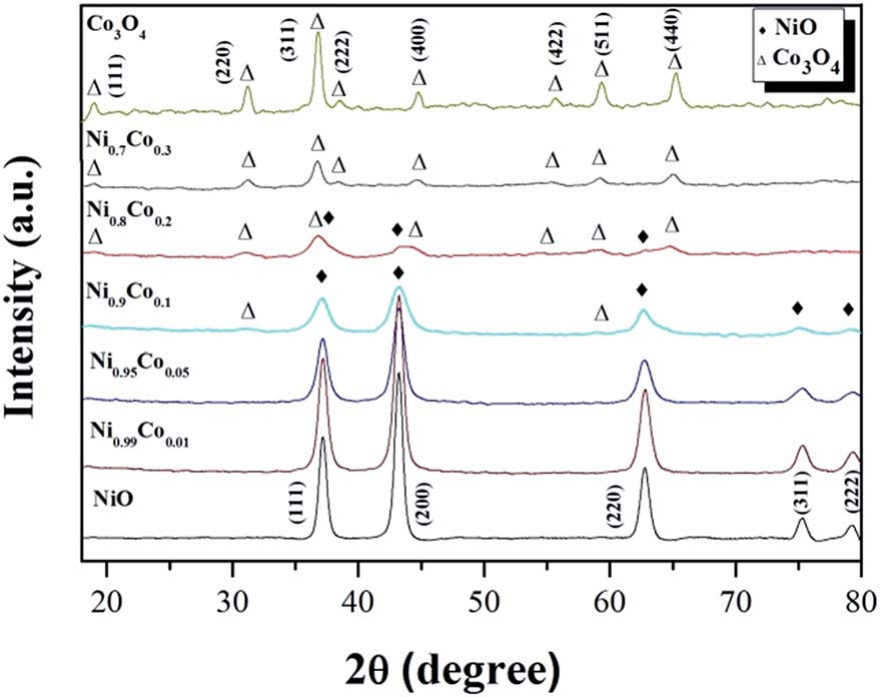
The XRD patterns of the as-prepared samples.

The XRD patterns of the Ni–Co composite oxides and the reference samples NiO and Co_3_O_4_ are shown in Fig. 2. From the reference patterns, the diffraction peaks at 37.2°, 43.3°, 62.9°, 75.4° and 79.4° are assigned to crystallographic planes (111), (200), (220), (311) and (222), respectively, of the face-centered cubic structure of NiO (JCPDS# 47-1049). The diffraction peaks at 19.0°, 31.3°, 36.8°, 38.8°, 44.8°, 55.7°, 59.4° and 65.2° are assigned to crystallographic planes (111), (220), (311), (222), (400), (422), (511) and (440), respectively, of the cubic spinel structure of Co_3_O_4_ (JCPDS# 42-1467). After the Co doping and when the Co content reaches 16%, only the diffraction peaks of the NiO phase were observed, indicating that no crystallized cobalt species were isolated from the NiO. Some possible reasons for the absence of cobalt diffraction peaks: (1) Co ions are incorporated into the nickel lattice; (2) Co particles are very small and highly dispersed, and therefore difficult to detect with XRD.^10,27^ However, the first argument can be ruled out since no shift of the diffraction peaks is observed, which would be expected with a modification of the NiO crystal lattice by incorporation of Co. By increasing the Co content, the diffraction peaks ascribed to the NiO phase become wider and weaker which is likely due to a gradual decrease in the crystallite size of the nickel oxide,^28^ as is also suggested by the calculated crystallite sizes presented in Table 1. When the Co content reaches 29%, diffraction peaks from the Co_3_O_4_ phase appear and the NiO phase peaks disappear. It indicates that Co_3_O_4_ crystals were formed and coexist with the NiO phase in the catalyst. Since the NiO reflections appear weaker and broader when the Co content is increased, this may indicate that the NiO loses its crystalline character. For the Ni_0.8_Co_0.2_ sample, only the diffraction peaks of spinel Co_3_O_4_ phase can be observed, suggesting that only small amounts of crystalline NiO is present in the catalyst. As mentioned before, the introduction of Co into NiO can effectively reduce the size of NiO, resulting in high dispersion of the metal oxide particles. We, therefore, deduce that amorphous NiO are formed and highly dispersed on the Co_3_O_4_ surface. Furthermore, it is worth noting that there is a negative shift for the diffraction peaks of the Co_3_O_4_ phase in *x* = 0.2 and 0.3 samples, compared with those of pure Co_3_O_4_, suggesting that Ni^2+^ could partially substitute Co^3+^ and move into the Co_3_O_4_ crystal lattice, forming the Ni–Co spinel structure.^25,29–31^ The octahedral Co^3+^ is coordinated to 6 O atoms; when it is substituted by Ni^2+^, oxygen vacancies form to compensate for the loss of positive charges, thereby retaining an overall neutrality of charges.^31^

**Fig. 3:**
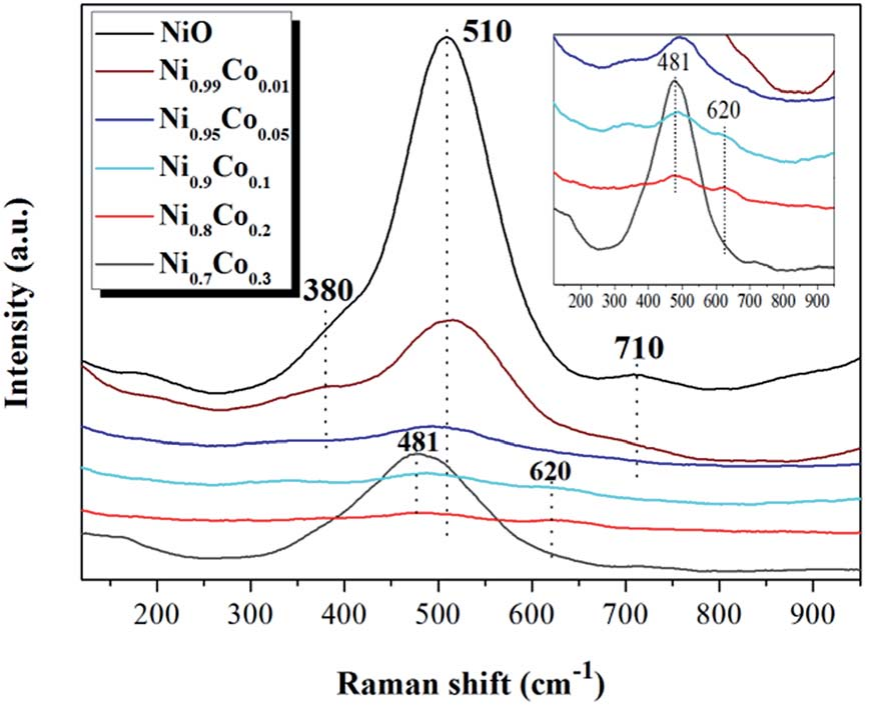
The laser Raman spectra of NiO and Ni–Co samples.

LRS was carried out to further investigate the influence of cobalt incorporation on the interior properties and surface structure of the Ni–Co samples. As shown in Fig. 3, the Raman spectra of pure NiO exhibits a main band at 510 cm^−1^ and a small band at 710 cm^−1^, corresponding to Ni–O stretching vibrational modes, and a shoulder peak at 380 cm^−1^, which is indicative of the non-stoichiometry of NiO.^32–34^ Compared with NiO, the intensity of the peaks from the Ni–Co samples become weaker and shift to lower frequencies, indicating that a strong interaction occurred between NiO and Co_3_O_4_ during preparation. Furthermore, when *x* ≥ 0.1, a broad peak is detected at 620 cm^−1^ which can be ascribed to surface oxygen vacancies, also related to the Frenkel defect-induced mode (D mode).^35^ It is interesting to note that the intensity of the peak at 481 cm^−1^ for the Ni_0.7_Co_0.3_ sample suddenly increased. According to the XRD results, this may be due to increased crystallinity of Co_3_O_4_. The band at 481 cm^−1^ can be assigned to vibrations of the spinel Co_3_O_4_.^36^

The textural information is summarized in Table 1. Relative to the data of pure NiO, the specific surface area, total pore volume and average pore diameter are all larger for the Ni–Co samples (with a few exceptions), which implies that the properties of the catalyst can be significantly modified with an appropriate amount of cobalt doping. Interestingly, the data exhibits a “Bell shape” as the cobalt content increases. The specific surface area increases from 112 m^2^ g^−1^ for the pure NiO sample to 135 m^2^ g^−1^ for the Ni_0.95_Co_0.05_ sample, likely due to a reduction in the size of the crystallites. As the cobalt content continues to increase, the crystal phase forms for Co_3_O_4_, and the specific surface area declines to 83 m^2^ g^−1^ for the Ni_0.7_Co_0.3_ sample, as supported by XRD results. Co_3_O_4_ possesses the smallest specific surface area (61 m^2^ g^−1^). Moreover, despite the Ni_0.95_Co_0.05_ sample having the largest specific surface area, it does not demonstrate optimal activity, suggesting that surface area is not a primary factor influencing catalytic behaviour. The Ni_0.8_Co_0.2_ sample possesses the largest average pore diameter, a relatively high specific surface area and total pore volume, and a unique mesoporous structure, and overall these contribute to enabling the compound to demonstrate the most effective catalytic activity. Moreover, from the N_2_-physisorption analysis, it can be seen that the Ni–Co samples are mesoporous (2–50 nm) structure, and their adsorption capacities are higher than NiO. The N_2_ adsorption–desorption isotherms (Fig. S3†) and corresponding analyses are presented in the ESI.†

**Fig. 4:**
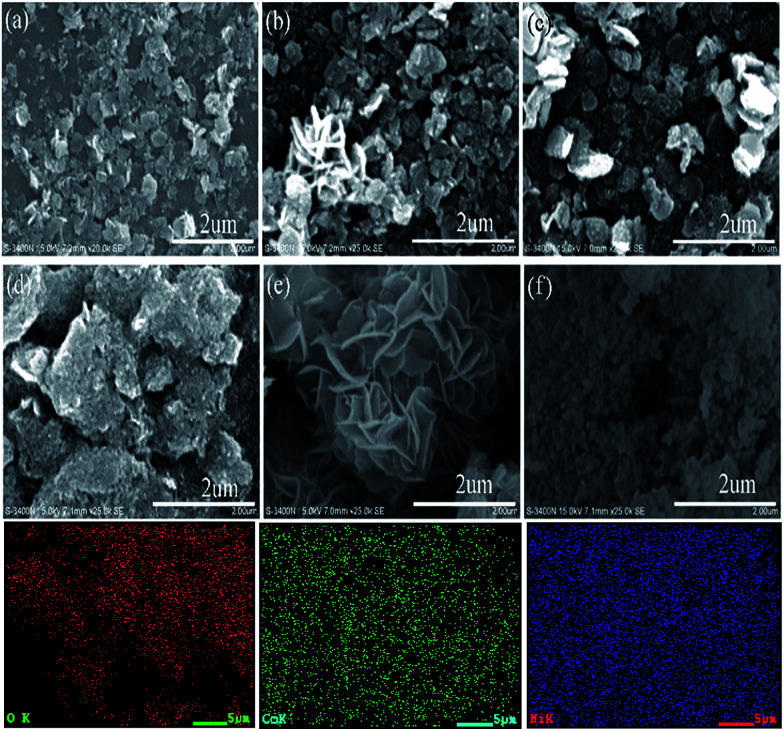
Typical SEM images of (a) NiO, (b) Ni_0.99_Co_0.01_, (c) Ni_0.95_Co_0.05_, (d) Ni_0.9_Co_0.1_, (e) Ni_0.8_Co_0.2_, and (f) Ni_0.7_Co_0.3_ samples; the EDS mapping images of Ni_0.8_Co_0.2_ sample are placed at the bottom.

SEM analysis was employed to observe the morphology of the NiO and Ni–Co samples. As depicted in Fig. 4, the chemical composition of the catalyst influences the morphology. For 0 ≤ *x* ≤ 0.05, the samples are uniformly small and plate-like with sizes between 300 to 500 nm. For *x* = 0.1, the sample remains plate-like but the plate sizes are larger and irregular. Interestingly, when *x* = 0.2, the catalyst morphology changed to “flower-like”, whereas, it became dense granules with a mean size of 70 nm for values of *x* up to 0.3. This variation of catalyst morphology may be associated with the sudden drop in specific surface area for the Ni_0.9_Co_0.1_ and Ni_0.7_Co_0.3_ samples. The unique flower-like morphology of the Ni_0.8_Co_0.2_ sample likely leads to distinctive textural properties, enabling it to exhibit excellent catalytic activity in the CO oxidation reaction. Elemental mapping analysis of SEM provides an intuitionistic elemental distribution of the Ni_0.8_Co_0.2_ sample, and proves the uniform distribution of Ni, Co and O in the sample. This result clearly indicates that the Ni species is highly dispersed, although there is an enrichment of Ni on the surface, which is consistent with the XRD and XPS results.

**Fig. 5:**
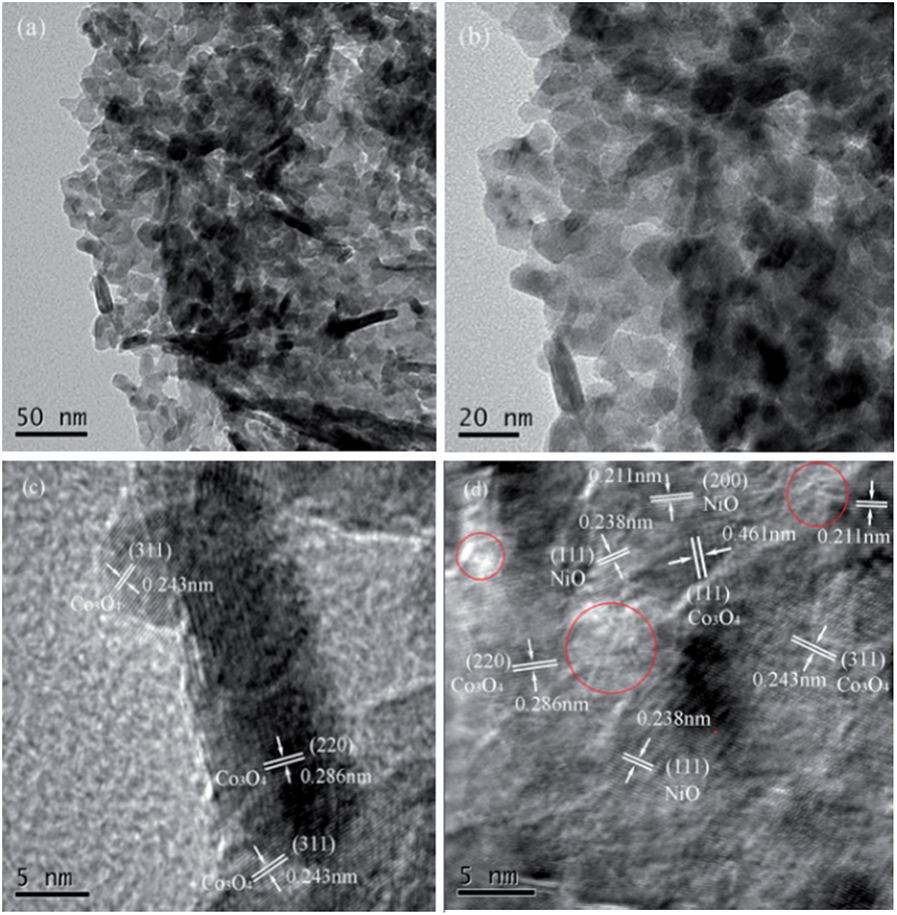
The morphology and microstructure of TEM (a, b) and HRTEM (c, d) images of Ni_0.8_Co_0.2_ sample.

**Fig. 6:**
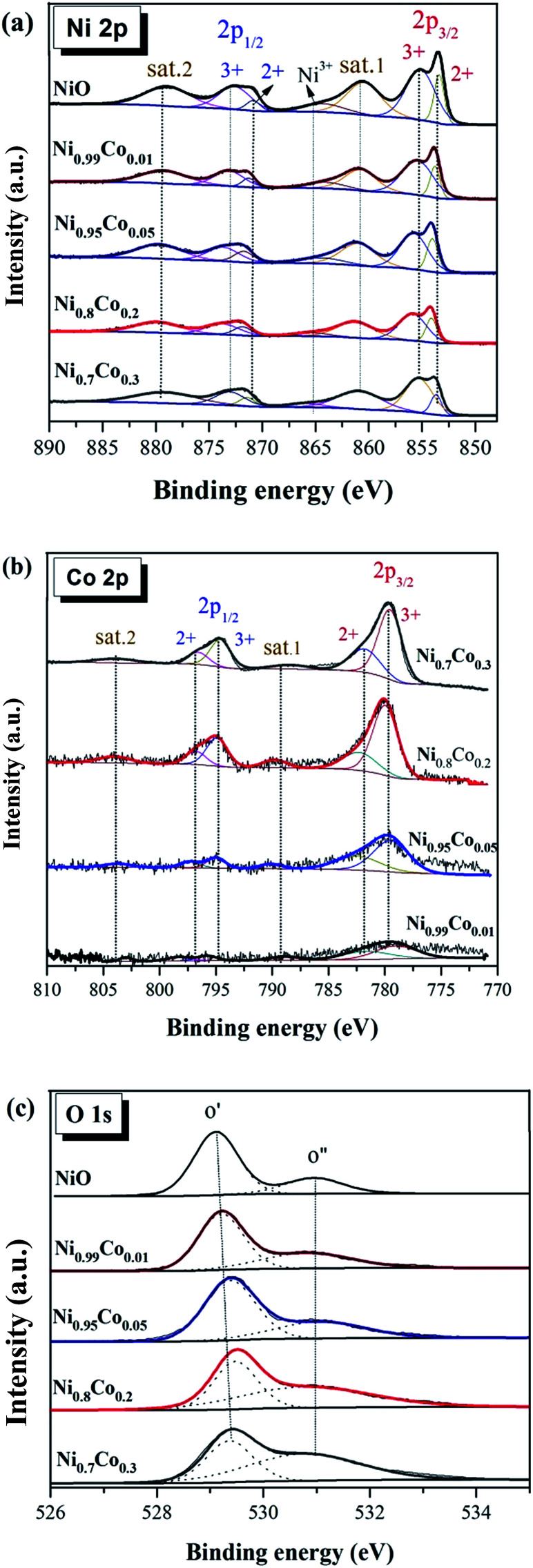
The XPS spectra of (a) Ni 2p, (b) Co 2p, (c) O 1s of several selected as prepared samples.

TEM and HRTEM images of the Ni_0.8_Co_0.2_ sample are shown in Fig. 5. It is made up of agglomerated particles with irregular shapes and sizes. The HRTEM images reveal the crystalline nature of the sample. Both bulk Co_3_O_4_ crystallites and tiny NiO crystallites can be observed from Fig. 6(d), as evidenced by the interplanar spacings of 0.461, 0.286, 0.243 and 0.238, 0.211 nm that correspond to the (111), (220), (311) planes of Co_3_O_4_ and the (111), (200) planes of NiO, respectively, which conforms to the XRD results. In addition, it can be noted that there is an amorphous phase marked by red circle in Fig. 6(d), which is highly likely to be amorphous NiO.

### 3.3 Surface chemical compositions analysis (XPS results)

XPS measurements were performed to explore the surface composition and elemental valence configuration of several selected samples, and the results are displayed in Fig. 6. The surface composition of these samples as calculated by XPS data are summarized in Table 2. Moreover, the binding energy values calibrated by C 1s (284.8 eV) allow for some error associated with charging effects during XPS analysis.

**Table 2:** The surface compositions of the several selected samples

Samples	Atomic concentration and atomic ratio by XPS							
	Atomic concentration (at%)				Atomic ratio (at%)		Ni/Co atomic ratio	
	C	Ni	Co	O	Co^3+^/(Co^3+^ + Co^2+^)	O′′/(O′′ + O′)	Bulk	Surface
NiO	6.37	50.21	—	43.42	—	24.45	—	—
Ni_0.99_Co_0.01_	12.03	43.18	0.4	44.79	57.49	35.66	28.57	107.95
Ni_0.95_Co_0.05_	12.75	38.78	3.9	44.53	63.03	33.02	5.13	9.94
Ni_0.8_Co_0.2_	15.84	29.53	8.53	46.1	72.36	56.91	1.29	3.46
Ni_0.7_Co_0.3_	17.40	21.47	12.69	48.44	62.67	60.96	0.82	1.69

Fig. 6(a) shows the XPS spectra of Ni 2p. The spectra from all as-prepared samples are similar and consist of two spin–orbit doublets and two shake-up satellites (denoted as sat.1 and sat.2). The first doublet at ∼853.8 and ∼871.5 eV and the second doublet at ∼855.8 and ∼873.2 eV are assigned to Ni^2+^ and Ni^3+^, respectively.^23,31,33,37^ The two intensive shake-up satellites (∼861.0 and ∼879.6 eV) are usually observed for paramagnetic Ni^2+^, and arise from charge transfer multi-electron transitions.^38,39^ In addition, the small peak at ∼865.0 eV is assigned to the shake-up satellite of Ni^3+^.^33,40^ It is clear that the proportion of this shake-up satellite for Ni_0.8_Co_0.2_ is smaller than that of other samples, suggesting the diminution of Ni^3+^ in the Ni_0.8_Co_0.2_ sample. This reveals that an intimate electronic transfer between nickel and cobalt may have occurred, which can be expressed as: Ni^3+^ + Co^2+^ → Ni^2+^ + Co^3+^. In general, the XPS results of Ni 2p suggest the formation of a defective NiO structure on the catalyst's surface, and the satellite peaks indicate that Ni^2+^ is the major component.

Fig. 6(b) shows the XPS spectrum of Co 2p. Four main peaks can be seen at ∼780, ∼795, ∼782 and ∼797 eV, and are assigned to Co^3+^ 2P_3/2_, Co^3+^ 2p_1/2_, Co^2+^ 2p_3/2_ and Co^2+^ 2p_1/2_, respectively, with an energy difference of the spin orbit split of 15 eV. Thus, the Co atom in these samples has two valence states (octahedral Co^3+^ and tetrahedral Co^2+^), indicating the formation of Co_3_O_4_;^41^ this is in line with the XRD results. The relative percentage content of Co and Co^3+^ is presented in Table 3. According to the literature,^2,6,42,43^ the surface Co^3+^ ions present a favourable site for CO adsorption and oxidation. However, Ni_0.7_Co_0.3_ possesses the highest surface Co^3+^ content, although its activity declines as compared to Ni_0.8_Co_0.2_. This indicates that Co species are not the dominant active species for the CO oxidation reaction.

**Table 3:** The peak areas of H_2_-TPR profiles and the H_2_ consumption (mmol g^−1^) of as prepared samples

Samples	H_2_-TPR results							
	Peak (ε) area	Peak (α) area	Peak (β) area	Peak (γ) area	Total peak area	Theoretical H_2_ consumption (mmol g^−1^)	Actual H_2_ consumption (mmol g^−1^)	T/A
Ni_0.99_Co_0.01_	390.75	—	12 300.36	1553.20	14 244.31	13.45	13.50	1.00
Ni_0.95_Co_0.05_	471.93	830.32	9050.53	3419.55	13 772.33	13.92	13.05	1.07
Ni_0.9_Co_0.1_	727.86	1556.82	6952.89	4385.28	13 622.85	14.23	12.91	1.10
Ni_0.8_Co_0.2_	768.94	1724.64	6240.31	5901.31	14 635.2	14.85	13.78	1.08
Ni_0.7_ Co_0.3_	801.24	2907.85	4820.18	7342.01	15 871.28	15.16	15.04	1.01
NiO	—	—	13 579.17	—	13 579.17	13.39	12.82	1.04
Co_3_O_4_ (0.01 g)	—	2402.55	—	8659.63	11 062.18	16.61	15.93	1.04

The high-resolution spectrum of O 1 s of these samples in Fig. 6(c) is fitted with two peaks: the main peak O′ at ∼529.2 eV, which is ascribed to the characteristic lattice oxygen bonding to the metal cations, and the shoulder peak O′′ with the higher binding energy at ∼530.8 eV, which is attributed to the chemisorbed oxygen.^44^ It reveals that oxygen vacancies exist on the sample's surface, and the O is adsorbed onto the surface in the form of O_2_^−^ or O^−^ ions,^45^ also demonstrated by the LRS results. The ratio of the chemisorbed oxygen is quantified based on the area ratio of O′′/(O′′ + O′) for these samples (Table 2) and follows the order: NiO < Ni_0.99_Co_0.01_ < Ni_0.95_Co_0.05_ < Ni_0.8_Co_0.2_ < Ni_0.7_Co_0.3_. The NiO sample has the lowest chemisorbed oxygen content (24.45%), whereas the chemisorbed oxygen content of the Ni_0.8_Co_0.2_ sample (56.91%) is much larger than that of NiO. It indicates that the introduction of cobalt facilitates the formation of oxygen vacancies on the catalyst surface. Moreover, the surface Ni/Co atomic ratios of the Ni–Co samples are higher than the corresponding bulk values, suggesting that there is an enrichment of Ni species on the catalyst surface.

### 3.4 Redox behavior and desorption analysis (H_2_-TPR and O_2_-TPD)

**Fig. 7:**
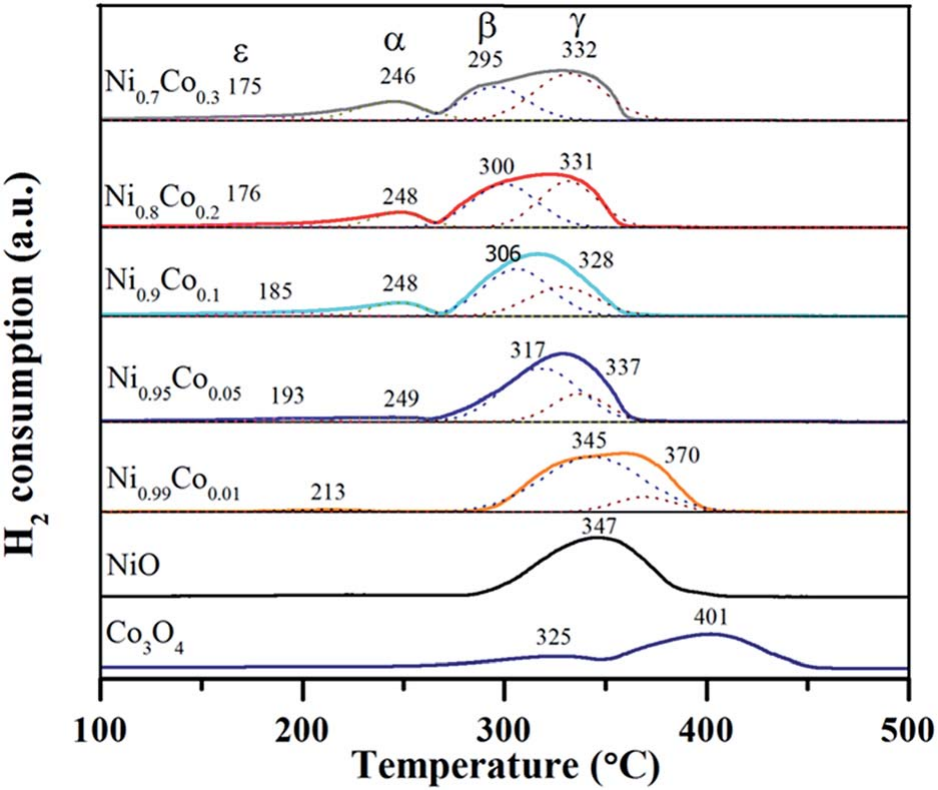
The H_2_-TPR profiles of as prepared samples.

H_2_-TPR characterization was performed to explore the reducibility of the samples and the interaction between NiO and Co_3_O_4_ on the Ni–Co catalysts, as shown in Fig. 7. The Co_3_O_4_ sample exhibited two reduction peaks at 325 and 405 °C, which correspond to the well-defined two-step reduction of Co^3+^ to Co^2+^ and Co^2+^ to Co^0^.^13,28,46^ For the NiO sample, only one broad peak at 347 °C was observed. As reported,^5,10,47^ the reduction of pure NiO particles usually takes place at around 350 °C. Accordingly, this broad peak is ascribed to the reduction of the NiO particles. When it comes to the Ni–Co samples, the former small peak (ε) with the lowest reduction temperature (175–213 °C) belongs to the reduction of surface oxygen species adsorbed on oxygen vacancies.^13,33^ The position of peak (ε) shifts slightly to a lower temperature as the Co content increases, indicating that the surface oxygen species on the Ni–Co samples is similar. The peaks (α) and (γ) at ∼246 °C and ∼332 °C, respectively, are assigned to the two-step reduction of the Co ions. The peak (β) in the temperature range from 295 to 345 °C is likely associated with the reduction of the NiO particles. Tang *et al.*^35^ assigned the peak around 300 °C to the reduction of well-dispersed NiO interacting strongly with the Ni–Ce solid solution. According to this point and combined with the XRD and TEM results, we infer that the peak (β) at lower temperature (∼300 °C) can be attributed to the highly dispersed amorphous NiO phase interacting strongly with the Ni–Co spinel, which supports the existence of amorphous NiO on the catalyst. In addition, peak (α) cannot be observed from the spectra of the Ni_0.99_Co_0.01_ sample, which is possibly due to the fact that the content of cobalt is too low and therefore the amount of Co^3+^ is extremely low. The position of peak (β) shifts to lower reduction temperatures gradually, in sync with an increase in Co content, which can be explained as follows. Firstly, there is an enhanced intense synergistic effect between NiO and Co_3_O_4_ through Ni^3+^ + Co^2+^ → Ni^2+^ + Co^3+^, which makes the nickel species easier to reduce. Secondly, as deduced from the XRD and TPR results, the introduction of Co turns the crystalline NiO into amorphous NiO and enhances the dispersion of NiO particles, which also contributes to the reduction of NiO particles. The positions of peak (α) and peak (γ) also shift towards lower reduction temperatures, which can be likened to pure Co_3_O_4_, likely due to the strong synergistic effect and the accumulation of Co^3+^ on the catalyst surface.

Detailed data of the reduction peak areas are displayed in Table 3. By comparing the peak area, it is clear that the area of peak (α) and peak (γ) increase as the Co content increases. However, the peak (β) area shows the opposite variation tendency. This suggests that the Co_3_O_4_ increases as the NiO decreases, which is in agreement with the XRF and XRD results. The peak (ε) exhibits the same trend with peak (α) and peak (γ), implying that doping with cobalt facilitates the generation of surface oxygen species. This trend agrees with the proportion of surface oxygen species within the Ni–Co catalysts (higher Co doping results in larger amount of surface oxygen) from the XPS results. In addition, the H_2_ consumption was calculated and is listed for each catalyst in Table 3. It can be seen that the ratios of theoretical H_2_ consumption (denoted as T) to actual H_2_ consumption (denoted as A) are slightly larger than 1.0, indicating that most of the H_2_ consumption come from the reduction of NiO. Furthermore, low valence Co^2+^ exists in the samples, also causing a decrease in the actual H_2_ consumption. This is in agreement with the XPS results.

**Fig. 8:**
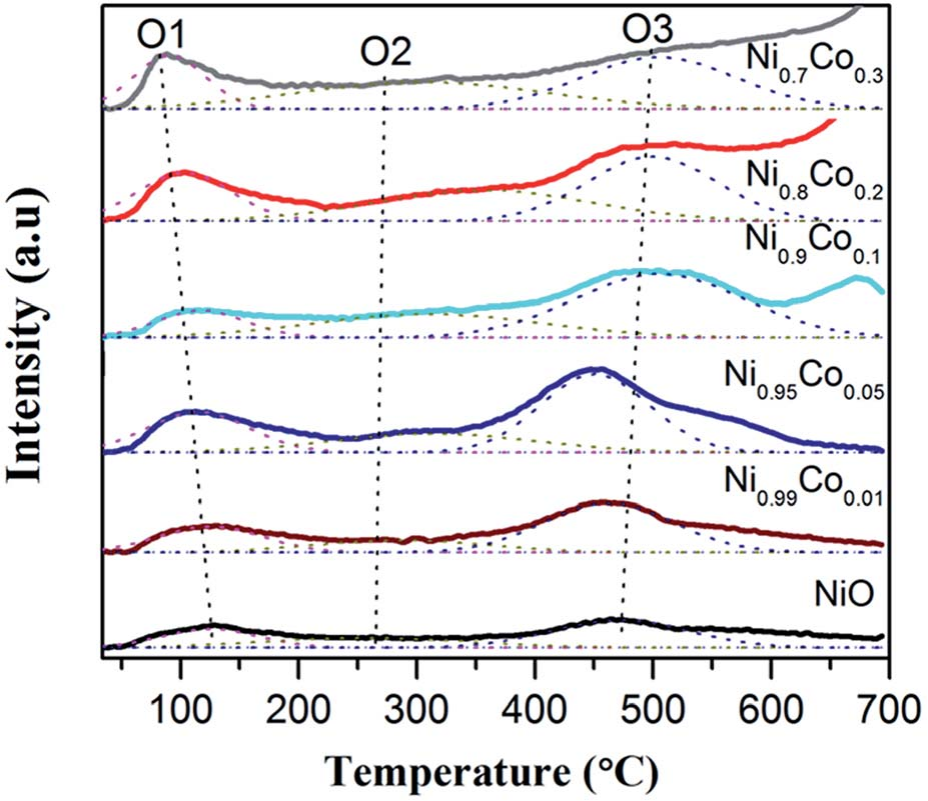
The O_2_-TPD profiles of pure NiO and Ni–Co samples.

O_2_-TPD experiments were carried out to further investigate how the surface oxygen species possibly affects the redox chemistry of the catalysts. According to the literature,^45^ desorption peaks below 400 °C usually belong to superficial oxygen species and are weakly bound to the surface. From Fig. 8, it can be noted that each sample has three desorption peaks at about 100, 325 and 495 °C. The first intense peak (O1) is ascribed to physically adsorbed oxygen species faintly bound to the surface, which is easily desorbed, even in a low temperature range. The second broad weak peak (O2) is attributed to the O_2_^−^ or O^−^ species, formed by the oxygen adsorbed on the surface vacancies; this corresponds well with the XPS results.^44,48^ The peaks above 450 °C are associated with the surface lattice oxygen species,^31^ which have nothing to do with the reaction due to their relatively high desorption temperatures. The first two peaks should be further analysed since they may be closely related to the oxidation and redox reactions.

The oxygen-supplying ability depends on the number of oxygen-supplying centers and the activity.^49^ Data for the former two peaks is displayed in Table 4. We can see that the area of peak O1 is enhanced compared with pure NiO with an addition of Co. It is interesting to note that the area of peak O1 is sequentially consistent with the specific surface area, indicating that a large specific surface area is beneficial to the physical adsorption of oxygen.^45^ It is also clear that the amount of adsorbed O_2_^−^ or O^−^ species for Ni–Co oxides increases remarkably compared with pure NiO.

**Table 4:** The peak areas of O_2_-TPD profiles pure prepared samples

Samples	O2-TPD results	
	Peak (O1) area	Peak (O2) area
Ni_0.99_Co_0.01_	1129.85	897.93
Ni_0.95_Co_0.05_	1674.72	1573.05
Ni_0.9_Co_0.1_	998.87	2253.28
Ni_0.8_Co_0.2_	1636.10	2480.52
Ni_0.7_Co_0.3_	1539.24	2702.76
NiO	752.85	846.18

### 3.5 CO and/or O_2_ interaction with NiO and Ni_0.8_Co_0.2_ samples (*in situ* DRIFTS results)

In order to gain more of an understanding about the nature of how the CO oxidation reaction occurs on the surface of the catalyst, the interactions between the reactants on the samples and the changes of the surface adsorbed species need to be examined. Therefore, the CO and/or O_2_ adsorption *in situ* DRIFTS spectra of the NiO and Ni_0.8_Co_0.2_ samples were recorded under simulated reaction conditions with temperatures ranging from 50 to 150 °C.

#### 3.5.1 Single CO adsorption on the NiO and Ni_0.8_Co_0.2_ samples

**Fig. 9:**
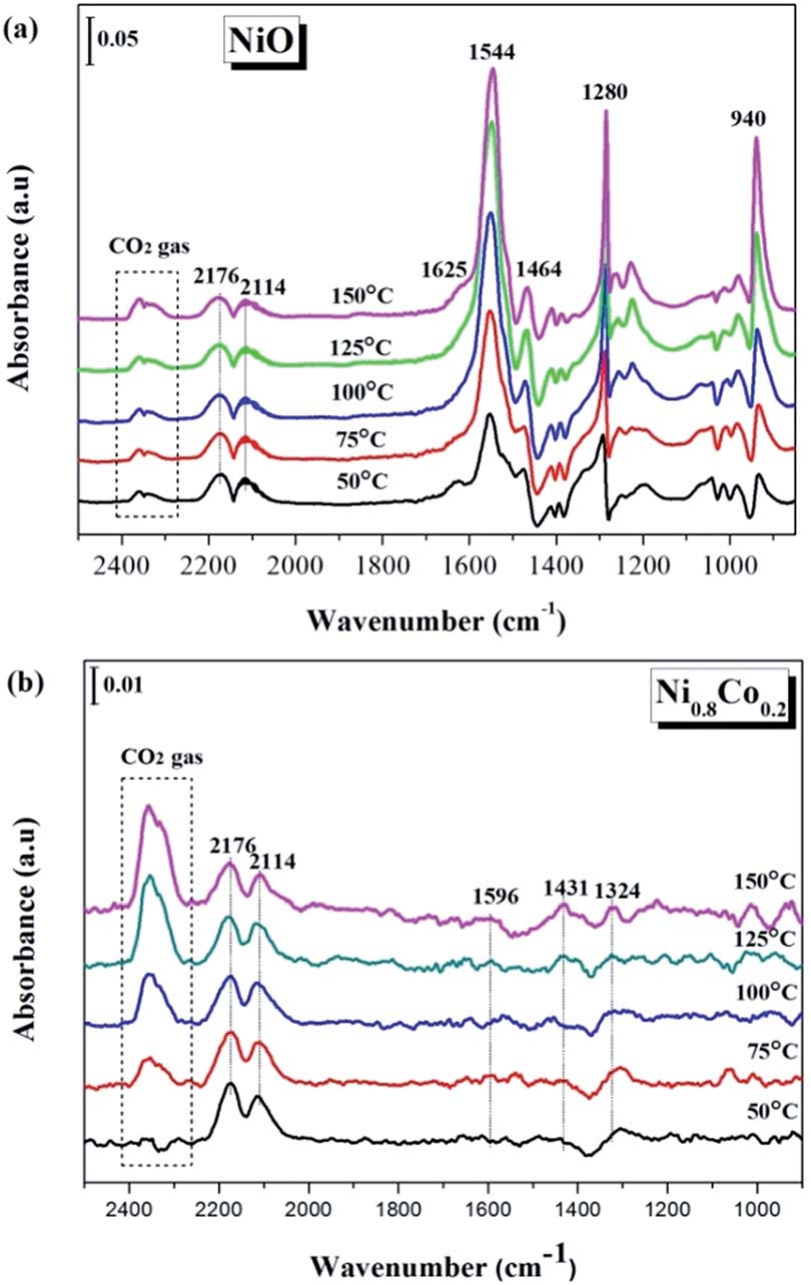
*In situ* DRIFTS spectra of (a) NiO and (b) Ni_0.8_Co_0.2_ samples under CO stream at different temperature.

Fig. 9 shows the DRIFTS spectra of CO adsorption on the NiO and Ni_0.8_Co_0.2_ samples, obtained *in situ*. The peaks at around 1625 and 1362 cm^−1^ are assigned to the bidentate bicarbonate and bidentate formate,^50^ respectively; the peaks at 1544, 1464 and 940 cm^−1^ can be attributed to surface carbonate species;^44,50,51^ and the peak around 1280 cm^−1^ is associated to carboxylate species^51^ (Fig. 9a). It is observed that the intensity of carbonate and formate species decreases as the temperature increases. According to Han *et al.*^27^ and Glisenti *et al.*,^42^ the bands detected at 2176 and 2114 cm^−1^ are attributed to gaseous CO the two peaks at 2330 and 2356 cm^−1^ appear simultaneously and therefore originate from gaseous CO_2_.

The *in situ* DRIFTS spectra of CO adsorption on the Ni_0.8_Co_0.2_ sample are shown in Fig. 9b. Peaks belonging to carbonate and formate species can also be detected at low temperatures; these peaks increase in intensity with a further rise in temperature. The bands ascribed to gaseous CO and gaseous CO_2_ are also observed at similar wavenumbers to the bands observed for the NiO sample (Fig. 9a). Moreover, the peak intensity of gaseous CO gradually reduces whilst the peak intensity of gaseous CO_2_ increases as the temperature increases, which is most likely a result of the catalyst reduction,^45^ suggesting that the Ni_0.8_Co_0.2_ sample is much easier to be reduced compared to the NiO sample.

#### 3.5.2 CO and O_2_ co-adsorption on the NiO and Ni_0.8_Co_0.2_ samples

**Fig. 10:**
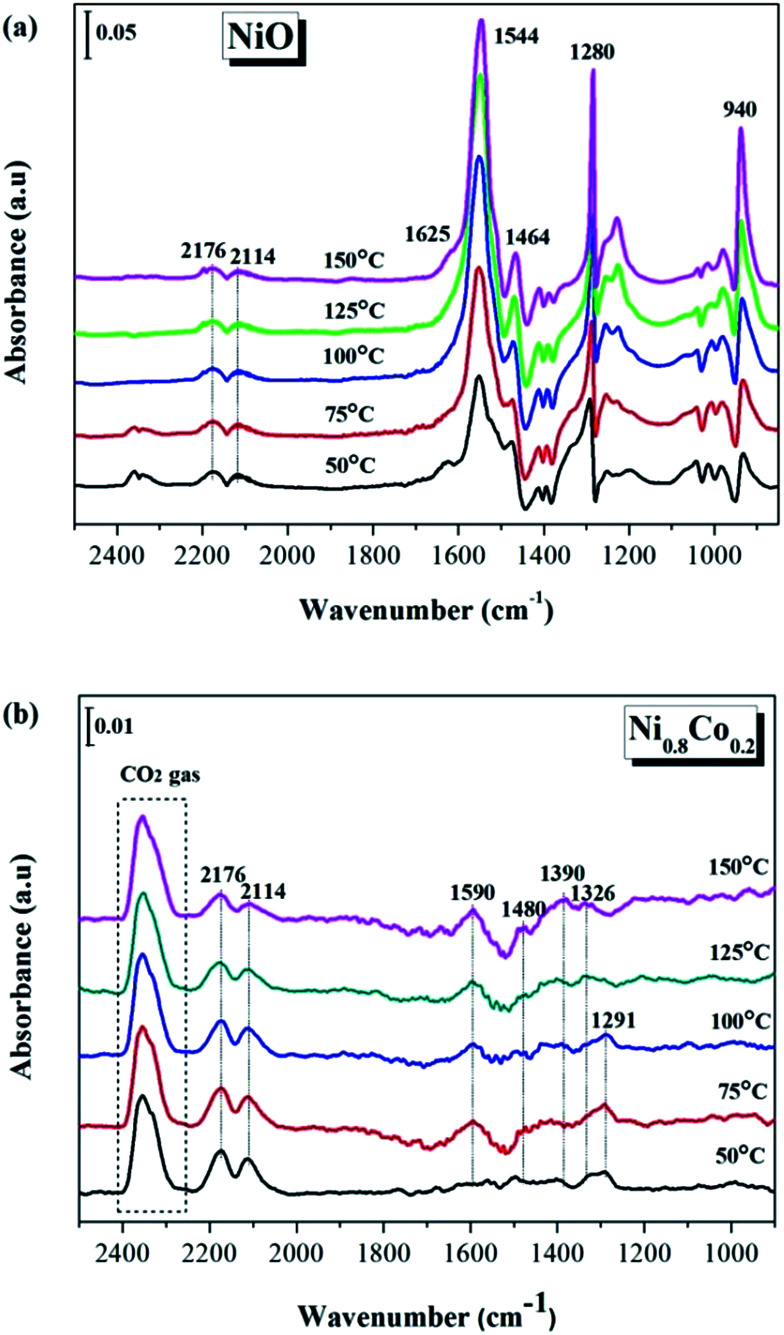
*In situ* DRIFTS spectra of (a) NiO and (b) Ni_0.8_Co_0.2_ samples under CO + O_2_ stream at different temperature.

In order to further investigate the nature of the surface reaction mechanism, the *in situ* DRIFTS spectra of CO and O_2_ co-adsorption were obtained under the simulated CO + O_2_ reaction conditions, as shown in Fig. 10. For both samples, a similar result is observed to what was described in the previous section (Fig. 9): the bands attributed to the various carbonate species formed by adsorbed CO molecules on the surface also appear in the range of 950–1700 cm^−1^. Furthermore, the peaks at 2114, 2176 cm^−1^ and 2330, 2356 cm^−1^ arise from gaseous CO and gaseous CO_2_, respectively. However, for the Ni_0.8_Co_0.2_ sample, the peaks of the intermediates are stronger than that of the CO adsorption, but their intensity is still relatively weak. Interestingly, the peaks of gaseous CO are weaker than that of the CO adsorbed on Ni_0.8_Co_0.2_ sample. This is likely due to the fact that the O_2_ molecules are preferentially adsorbed on the sample surface in an oxygen-enriched atmosphere, forming surface-active O species, therefore inhibiting the accumulation of CO.^49,52^ Furthermore, the band for gaseous CO_2_ (2330 and 2356 cm^−1^) exhibits a distinct increase compared to Fig. 10(b), suggesting that oxygen accelerates the reaction rate.

It is worth noting that for the NiO sample a large number of carbonate and carboxylate species are generated during the CO or/and O_2_ adsorption processes, while the Ni_0.8_Co_0.2_ sample shows the opposite behaviour. It is generally accepted that monometallic Ni catalysts are prone to carbon formation, which usually causes catalyst deactivation.^24,53^ Therefore, the following can be concluded: in the case of the NiO sample, the CO molecules were oxidized by surface active O species, initially forming a large number of carbonate and carboxylate species. These were deposited on the NiO surface but only a few of each species was converted to CO_2_ at low temperatures; therefore it was these that covered the active sites on the surface. On the contrary, the Ni_0.8_Co_0.2_ sample possesses many surface oxygen vacancies and therefore only a few carbonate species were generated on the surface under the simulated reaction conditions, indicating that CO molecules can rapidly be oxidized by surface-active O to CO_2_ gas. The oxygen vacancies therefore need to be fully exposed to support an efficient and stable CO oxidation reaction. Ren *et al.*^31^ have also reported that doping of Ni in Co_3_O_4_ reduces the formation of stable carbonates on the catalyst surface, which promotes the desorption of CO_2_ during the oxidation of propane.

**Fig. 11:**
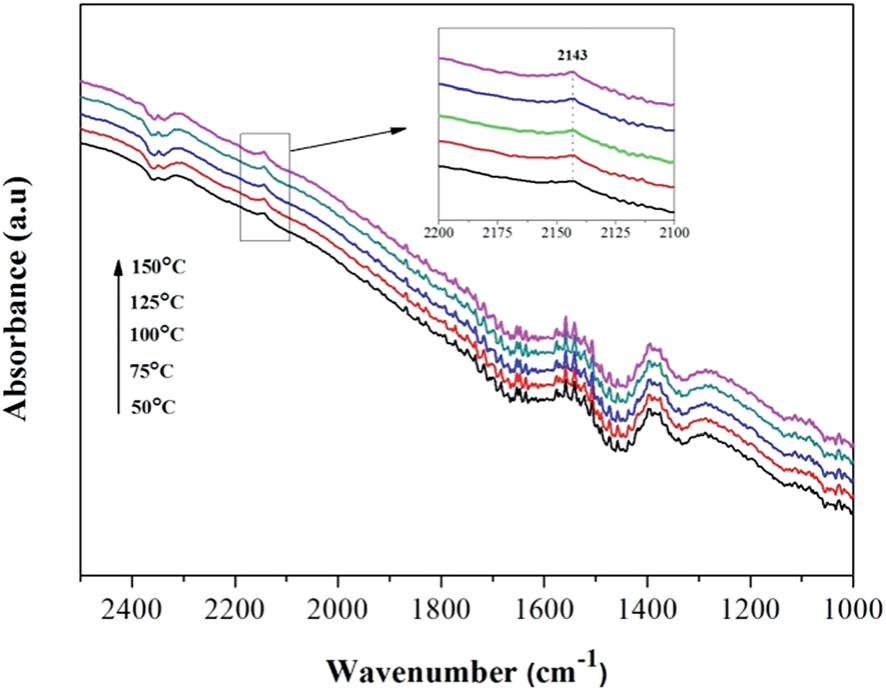
*In situ* DRIFTS spectra of Ni_0.8_Co_0.2_ sample under CO + O_2_ stream after subtracting the contribution of gaseous CO in the DRIFTS cell.

Amorphous NiO has been recognized as being active for CO oxidation with CO molecules being adsorbed by Ni^2+^ to form of Ni^2+^–(CO) and Ni^2+^–(CO)_2_.^9^ However, the FT-IR signals of these complexes are typically too weak to be detected or they overlap with the signal for gaseous CO. To understand whether surface–adsorbed CO exists on the Ni_0.8_Co_0.2_ catalyst surface under reaction conditions, the reference of gaseous CO was measured in the DRIFTS cell and the contribution subtracted (the results are shown in Fig. 11). It is interesting to notice the appearance of a small peak at 2143 cm^−1^. Solsona *et al.*^33^ assigned a band at 2148 cm^−1^ to adsorbed CO on pure NiO. Hence, we inferred this small peak should be attributed to surface–absorbed CO on the surface Ni species. The peak intensity increases as the temperature increases, indicating that higher temperatures are favourable to the adsorption of CO. Furthermore, the single CO adsorption *in situ* DRIFTS spectra of Ni_0.8_Co_0.2_ and NiO was compared after subtracting the contribution of gaseous CO. Fig. S4† shows that the intensity of the adsorbed CO band of Ni_0.8_Co_0.2_ is stronger than that of NiO, indicating that Ni_0.8_Co_0.2_ has a stronger CO adsorption.

### 3.6 The possible mechanism for CO oxidation reaction over Ni–Co–O catalysts

As far as catalyst development is concerned, it is critical to explore the structure–activity correlation of catalysts. To the best of our knowledge, little has been reported about the reaction mechanism of CO oxidation catalysed by nickel–cobalt catalysts. Based on the information from previous characterization, especially the CO or/and O_2_ adsorption *in situ* DRIFTS results, a mechanism for CO oxidation reaction is tentatively proposed. Considering the activity test results, cobalt doping is required for the most effective catalytic activity. Combining the results of XRD and H_2_-TPR, it can be concluded that a low cobalt content does not result in a high dispersion of the NiO particles, leading to a low activity. On the other hand, a large cobalt content can reduce the surface NiO concentration and the specific surface area, resulting in a decrease of activity. Therefore, it can be deduced that highly dispersed surface amorphous NiO is the dominant active species, similar to the study performed by Tang *et al.*^10,35^ By comparing the *in situ* DRIFTS results of NiO and Ni_0.2_Co_0.8_ samples, it was deduced that a high concentration of oxygen vacancies play an important role in the CO + O_2_ reaction, which is supported by Raman, XPS and O_2_-TPD results. Mahammadunnisa *et al.*^54^ fabricated NiO/Ce_1−*x*_Ni_*x*_O_2−*δ*_ catalysts for CO oxidation and observed that an enrichment of oxygen vacancies on the surface of the catalyst can promote the activation of oxygen species on surface and accelerate the reaction.

**Fig. 12:**
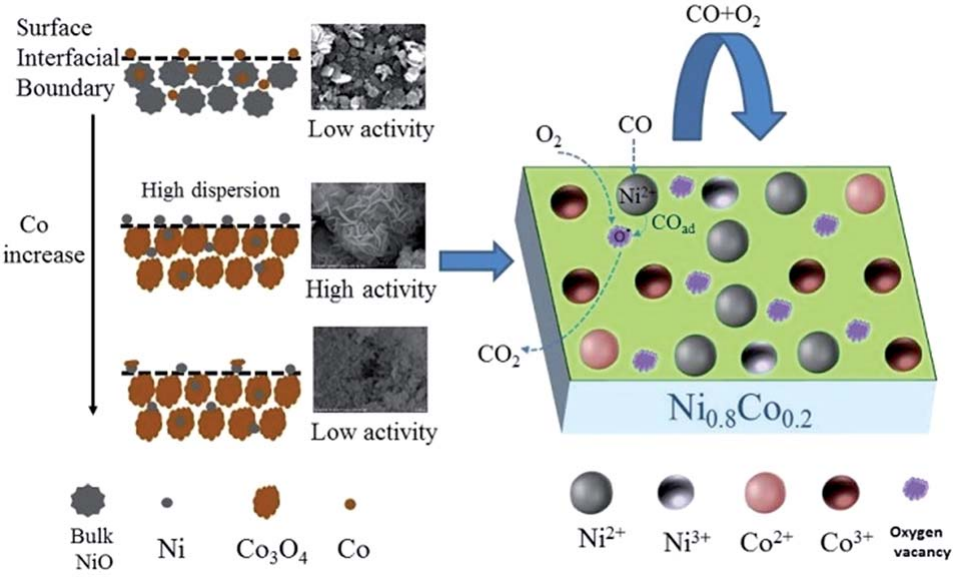
The possible reaction mechanism for CO oxidation over the Ni_0.8_Co_0.2_ sample.

Based on the above results, when the surface–adsorbed CO reacts with activated O over the Ni–Co samples, it does so according to a Langmuir–Hinshelwood (L–H) mechanism. As depicted in Fig. 12 and taking the Ni_0.8_Co_0.2_ sample as an example, O_2_ molecules preferentially adsorb on the catalyst surface in an oxygen-enriched atmosphere, forming surface-active oxygen species (such as O_2_^−^ or O^−^), which occupy surface vacancies. CO molecules are adsorbed on the surface NiO (amorphous) to form Ni^2+^–CO species, and the adsorbed CO then reacts with the active oxygen species on nearby surface oxygen vacancies and is transformed into gaseous CO_2_. Finally, the surface oxygen vacancies are regenerated by gaseous O_2_, completing the catalytic cycle.^55^

## 4. Conclusions

In this work, a series of Ni–Co composited oxide catalysts with different Ni/Co ratios were synthesised by a facile liquid-precipitation method and tested for their ability to catalyse the CO oxidation reaction. Based on the above characterization, results and discussion, several major conclusions were formulated:

(1) The doping of Co species to form binary composite oxides can effectively enhance the redox properties and catalytic activity of nickel oxide. The synergetic effect between Ni and Co leads to a significant decrease in the size of the NiO, resulting in the formation of highly dispersed amorphous NiO on the catalyst surface, which strongly reacts with Co_3_O_4_. The highly dispersed amorphous NiO is presumed to be the dominant active species for the CO oxidation. The direction of the redox equilibrium, expressed as Ni^3+^ + Co^2+^ → Ni^2+^ + Co^3+^, translates as the Ni–Co oxides being more easily reduced than pure NiO.

(2) The surface oxygen vacancies play an important role in the reaction atmosphere. For the Ni_0.8_Co_0.2_ sample, the combination of a high concentration of surface oxygen vacancies and the regeneration of oxygen vacancies leads to excellent catalytic activity and stability in the CO oxidation reaction.

(3) As the amount of cobalt increases, the morphology of the catalyst changes from plate-like to flower-like, and, eventually, to dense granules. The Ni_0.8_Co_0.2_ shows a novel flower-like morphology and demonstrates the best catalytic performance.

(4) O_2_ molecules can be translated into activating O species (O^2−^ or O^−^) through absorption by the surface oxygen vacancies. The surface-adsorbed CO reacts with the activating O species to produce CO_2_*via* a classic L–H reaction mechanism.

(5) The Ni–Co composite oxide exhibits higher catalytic activity than other Ni-based composite oxide including Ni–Mn, Ni–Fe, Ni–Zn and Ni–Cr.

## Conflicts of interest

There are no conflicts of interest to declare.

## Supplementary Material

RA-008-C7RA12635B-s001
