# Supplementary material for: Low temperature CO oxidation catalysed by flower-like Ni–Co–O: how physicochemical properties influence catalytic performance
Source: RSC Adv. 2018 Feb 12;8(13):7110–22. doi: 10.1039/c7ra12635b (PMC9078297; doi:10.1039/c7ra12635b)
Supplement: RA-008-C7RA12635B-s001 [file RA-008-C7RA12635B-s001.pdf]

Electronic Supplementary Material (ESI) for RSC Advances.
This journal is © The Royal Society of Chemistry 2018

# Low temperature CO oxidation over flower-like Ni-Co-O catalyst: the relationship between physicochemical properties and catalytic performance

Yunan Yi [a], Pan Zhang [a], Zuzeng Qin[a], Chuxuan Yu [a], Wei Li [a], Qiuju Qin [a], Bin Li [a,c,*], Minguang Fan[a], Xin Liang[a], Lihui Dong [a,b,*]

[a] *Guangxi Key Laboratory Petrochemical Resource Processing and Process Intensification Technology, School of Chemistry and Chemical Engineering, Guangxi University, Nanning 530004, PR China*

[b] *Jiangsu Key Laboratory of Vehicle Emissions Control, Center of Modern Analysis, Nanjing University, Nanjing 210093, PR China*

[c] *Guangxi Colleges and Universities Key Laboratory of Applied Chemistry Technology and Resource Development, Guangxi University, Nanning, 530004, PR China*

## Supplment data:

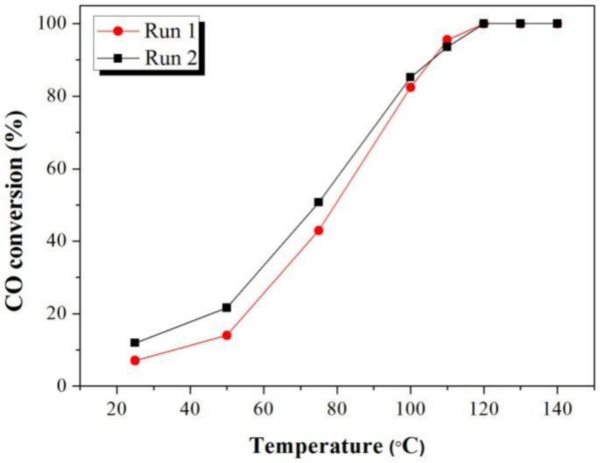


**Fig. S1** First and second run CO conversion with the reaction temperature of $Ni_{0.8}Co_{0.2}$ sample.

From Fig. S1, we can see that the CO conversion of second run is higher than that of first run at lower temperature (under 100 ℃), which may be result from the removal of adsorbed species impeding the adsorption of CO and/or oxygen.[1]

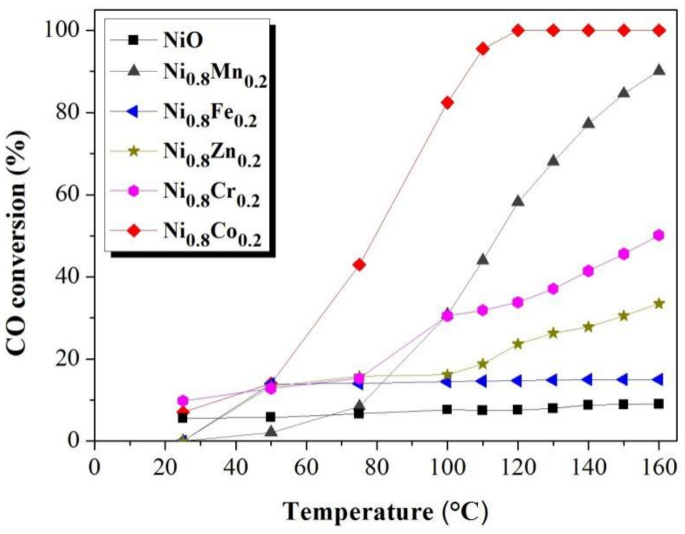


**Fig. S2** CO conversion (%) over $Ni_{0.8}M_{0.2}$ (M= Co, Mn, Fe, Zn, Cr) and NiO samples.

The activities of $Ni_{0.8}M_{0.2}$ (M = Mn, Fe, Zn, Cr, Co) composite oxides for CO oxidation are shown in Fig. S2, and pure NiO as a comparison. Upon doped with diverse metal elements, the activities elevated in varying degrees and follow the order as: $Ni_{0.8}Co_{0.2}$ > $Ni_{0.8}Mn_{0.2}$ > $Ni_{0.8}Cr_{0.2}$ > $Ni_{0.8}Zn_{0.2}$ > $Ni_{0.8}Fe_{0.2}$ > NiO. For the worst $Ni_{0.8}Fe_{0.2}$ sample, it exhibited similar performance as pure NiO and got the CO conversion of 15% at 160 °C, which only five percent higher than that of pure NiO. Except for $Ni_{0.8}Zn_{0.2}$ sample, the CO conversion values of other samples were elevated remarkably from 75 to 160 °C. Among all samples, the $Ni_{0.8}Co_{0.2}$ sample performed the most excellent activity, which made CO entirely convert at 120 °C. These results indicate that introduced $M^{x+}$ (M = Co, Mn, Fe, Zn, Cr,) to formed binary oxides can enhance the activity of the catalyst in varying degrees, and this effect of cobalt is the most significant.

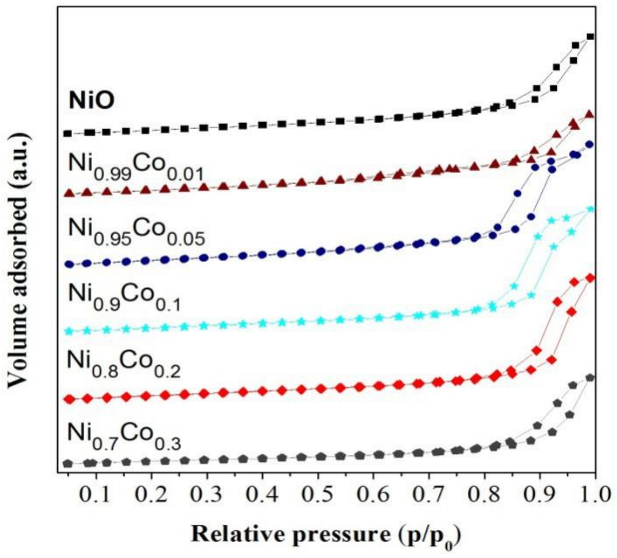


**Fig. S3** The $N_2$ adsorption-desorption isotherms for pure NiO and Ni-Co samples.

The $N_2$ adsorption-desorption isotherms of the as prepared samples are depicted in Fig. S3. From the figure, it can be seen that all samples exhibit type IV isotherms with evident H3-type hysteresis loops, indicative of their mesoporous (2-50 nm) structure which is typical for narrow slit-like shapes or plate-like particles, according to IUPAC.[2] For the six samples, their adsorption branches ascend slowly at low relative pressure range of $P/P_0 \leq 0.8$; but they exhibit a pronounced capillary condensation step beginning at about $P/P_0$ =0.8. The jumps at $P/P_0$ = 0.8-0.9 are ascribed to the capillary condensation of the mesopores produced by removal of silica walls, and the steeper jumps at $P/P_0$ = 0.9-1.0 can be attributed to the interstitial pores between particles.[3] Besides, the area of the hysteresis loop is related to the adsorption capacity of catalyst. We can see that the adsorption capacity of these samples increases initially and then decreases with the increase of Co content, its law of variation is roughly consistent with that of BET results and pore volume are summarized in Table 1.

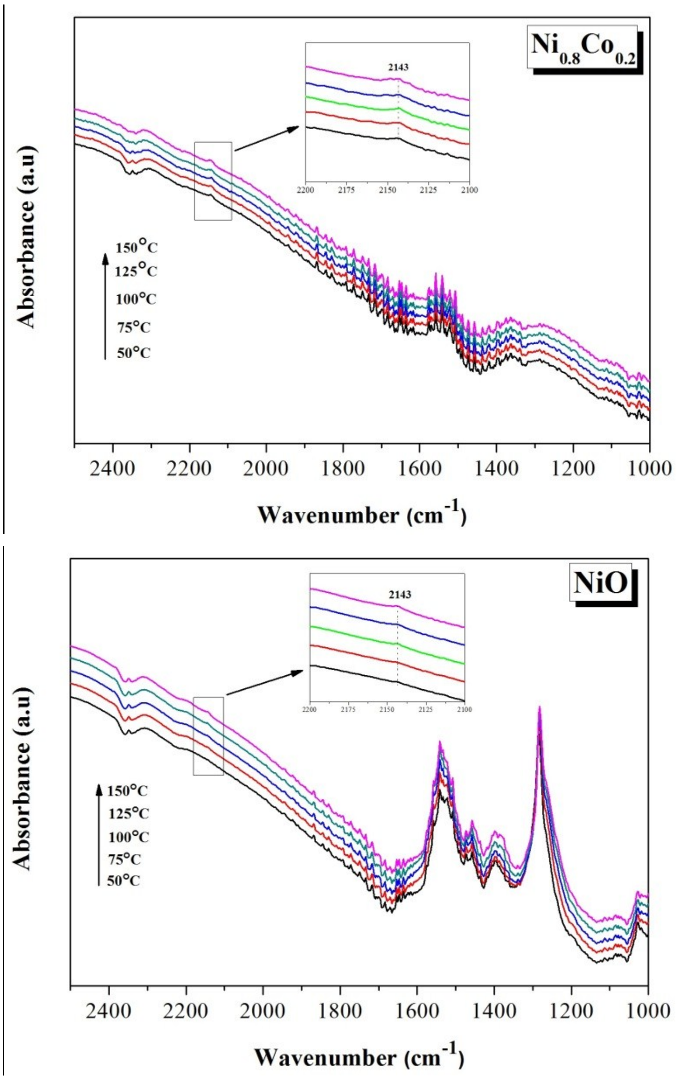


**Fig. S4** In situ DRIFTS spectra of $Ni_{0.8}Co_{0.2}$ and NiO samples under CO stream after subtracting the contribution of gaseous CO in the DRIFTS cell.

## References:


1 Y.I. Choi, H.J Yoon, S.K. Kim, Crystal-facet dependent CO oxidation, preferential oxidation of CO in $H_2$-rich, water-gas shift reactions, and supercapacitor application over $Co_3O_4$ nanostructures, Appl. Catal. A: Gen.,

2016, 519, 56-67.

2 C.S. Deng, B. Li, L.H. Dong, F.Y. Zhang, M.G. Fan, G.Z. Jin, J.B Gao, L.W Gao, F. Zhang, X.P. Zhou, NO reduction by CO over CuO supported on $CeO_2$-doped $TiO_2$: the effect of the amount of a few $CeO_2$, Phys. Chem. Chem. Phys., 2015, 17, 16092–16109.

3 C.J. Tang, J.C. Li, X.J. Yao, J.F. Sun, Y. Cao, L. Zhang, F. Gao, Y. Deng, L. Dong, Mesoporous NiO–$CeO_2$ catalysts for CO oxidation: Nickel content effect and mechanism aspect, Appl. Catal. A: Gen., 2015, 494, 77–86.
